# The Clinical COPD Questionnaire Correlated with BODE Index-A Cross-Sectional Study

**DOI:** 10.1100/2012/361535

**Published:** 2012-04-30

**Authors:** Shih-Feng Liu, Ching-Wan Tseng, Mei-Lien Tu, Chin-Chou Wang, Chia-Cheng Tseng, Chien-Hung Chin, Meng-Chih Lin, Jien-Wei Liu

**Affiliations:** ^1^Division of Pulmonary & Critical Care Medicine, Kaohsiung Chang Gung Memorial Hospital and Chang Gung University College of Medicine, Kaohsiung 833, Taiwan; ^2^Department of Respiratory Therapy, Kaohsiung Chang Gung Memorial Hospital and Chang Gung University College of Medicine, Kaohsiung 833, Taiwan; ^3^Division of Infectious Diseases, Department of Internal Medicine, Kaohsiung Chang Gung Memorial Hospital and Chang Gung University College of Medicine, Kaohsiung 833, Taiwan

## Abstract

The Global initiative for Chronic Obstructive Lung Disease (GOLD) staging has widely used in the stratification of the severity of COPD, while BODE (body mass index, airflow obstruction, dyspnea, and exercise capacity) index was proven superior to FEV1 in predicting mortality, exacerbation and disease severity in patients with COPD. Clinical COPD Questionnaire (CCQ), a questionnaire with ten items categorized into three domains (symptoms, functional state and mental state) was developed to measure health status of COPD patients. However, little is known about the relationship between CCQ score and BODE index. We performed a prospective study with the inclusion of 89 patients who were clinically stable after a 6-week-therapy for COPD symptoms comparing their health status assessed by CCQ, BODE index and GOLD staging. We found that the total CCQ score was correlated with BODE score (*P* < 0.001) and GOLD staging (*P* < 0.001); of three CCQ domains, the functional status correlated the most with BODE index (*rS* = 0.670) and GOLD staging (*rS* = 0.531), followed by symptoms (*rS* = 0.482; *rS* = 0.346, respectively), and mental status (*rS* = 0.340; *rS* = 0.236, respectively). Our data suggest that CCQ is a reliable and convenient alternative tool to evaluate the severity of COPD.

## 1. Introduction

Chronic obstructive pulmonary disease (COPD) is a disease state characterized by nonfully reversible airflow limitation. The airflow limitation is usually progressive and associated with an abnormal inflammatory response of the lungs to noxious particles or gases [1]. Conventionally, a forced expiratory volume in the first second (FEV1) and a low FEV1/forced vital capacity (FVC) ratio are used to grade the severity of COPD, and the Global Initiative for Chronic Obstructive Lung Disease (GOLD) definition for airflow limitation is a FEV1/FVC ratio of less than 70% [1]. However, the systemic manifestations of COPD were not necessarily fully reflected by FEV1/FVC in clinical practice [2–6]. BODE index, a multidimensional parameter including the body-mass index (B), the degree of airflow obstruction (O), functional dyspnea (D), and exercise capacity (E), was proposed by Celli et al. [2] and was reported to be superior to FEV1 in reflecting the severity of COPD and effective in predicting the mortality in patients with COPD [2]. BODE index was also reported to be applicable in predicting an individual's need for hospitalization [7], measuring follow-up lung functional change in pulmonary rehabilitation [8] as well as transbronchoscopic placement of one-way valves [9], predicting the survival after a patient receiving lung volume reduction surgery [10] and reflecting the disease modification [11].

Since 1948, when the World Health Organization defined health as being not only the absence of disease and infirmity but also the presence of physical, mental, and social well-being, quality of life has been considered ever increasingly important in healthcare practice and research [12]. Based on validated evidence [13–16], in 2003, the Clinical COPD Questionnaire (CCQ) was developed to measure health status of COPD patients [17–20]. The CCQ is a short health status questionnaire with ten items that are categorized into three domains, namely, symptoms, functional state, and mental state. The grading of measurements of CCQ was proven to be a function of changes in symptom severity in patients with COPD and in patients at risk for COPD, and reliable in discriminating between groups of patients that differ in severity of COPD [13]. One of the primary aims in the development of the CCQ was to create a scale capable of measuring changes in health status (e.g., changes of pre- and postintervention health status in the patients in question). The minimal clinically important difference of the total CCQ score is 0.4, which is used to judge whether a particular change in score represents a significant improvement or deterioration, or whether it represents a trivial change [21].

BODE index was reported to be good at predicting the worsening of health-related quality of life in COPD patients as measured by the St. George's Respiratory Questionnaire (SGRQ) of which the evaluation is limited to the subdomains of subjective symptoms and subjective impairment [22–24]. CCQ score helps clinicians identify not only the clinical status of the airway but also activity limitations and emotional dysfunction in the patients with COPD. However, little is known about the correlation of CCQ score and BODE index in terms of measurement of quality of life in the clinically stable COPD patients. This study aimed to elucidate this information by comparing the status of COPD patients measured by CCQ and those measured by 2 different schemes: the GOLD classification criteria and the BODE index.

## 2. Methods

### 2.1. Study Design

A prospective study was conducted to investigate the association between the Clinical COPD Questionnaire (CCQ) and COPD severity assessed by two criteria: the GOLD staging and the BODE index.

### 2.2. Study Subjects

Between August 2005 and July 2006, a total of 89 patients with a wide range of symptom severity of COPD were recruited into a prospective study from the outpatient clinic of the Division of Pulmonary Medicine, Kaohsiung Chang Gung Memorial Hospital, a 2300-bed facility serving as a primary care and tertiary referral center in Taiwan. This study was approved by the Institutional Review Board of the research hospital. Each recruited COPD patient (aged ≥ 40 years and with written consent) was a heavy smoker who had an at least 10-pack-year history of smoking. The diagnosis of COPD was made based on the GOLD criteria: FEV1/FVC < 0.7 and reversibility to inhaled bronchodilator in FEV1 < 15% [25]. Other causes of airflow limitation such as pulmonary tuberculosis, bronchial asthma, bronchiectasis, and heart failure were excluded in the enrolled patients by reviewing their chest radiographs and medical histories. After a 6-week appropriate therapy, the clinically stable COPD patients were included and subjected to calculation of BODE index and measurement with CCQ. Patients who were experiencing exacerbation of COPD (e.g., presence of fever, increased purulent sputum, and dyspnea) or were hospitalized for whatever reasons during the 6-week-therapy period were excluded from this study.

### 2.3. Assessment with BODE Index

The calculation of BODE index was carried out using an empirical model as previously described [2]. Specifically, the degree of airflow obstruction was measured by FEV1, dyspnea by the modified Medical Research Council (MMRC) dyspnea scale [26], and exercise capacity by the distances of the six-minute-walk test (6MWD) [27]. For each value of FEV1, MMRC dyspnea scale, 6MWD, the patients received points ranging from 0 to 3; for body mass index, the point was either 0 or 1 [2]. The points for each BODE component in each included patient were added, so that the BODE index ranged from 0 to 10 points for each individual. The BODE score was further quartilized as follows: quartile 1 (a score of 0 to 2 points), quartile 2 (a score of 3 to 4 points), quartile 3 (a score of 5 to 6 points), and quartile 4 (a score of 7 to 10 points) [2]. 

### 2.4. Assessment with CCQ

Measurement of health status of the included patients was performed using the copyrighted CCQ (see the appendix) [13], with the approval from Professor van der Molen Thys. The CCQ consists of ten items (questions), which are allocated in the following three domains: symptoms (items 1, 2, 5, and 6), functional state (items 7, 8, 9, and 10), and mental state (items 3 and 4). Each CCQ item in this study is presented as CCQn, where n is the number corresponding to the numeric order of the question in CCQ. The enrolled patients were asked to record their experiences in the seven days immediately before measurement of their health status in a week-version CCQ. The CCQ total score and the CCQ domain scores were derived from dividing the sum of graded points for each related question by the number of questions [13]. The CCQ total score and the CCQ domain scores varied between 0 (indicating very good health status) to 6 (indicating extremely poor health status). The CCQ was completed by each included patient at his outpatient visit and was returned to a study coordinator. To avoid confounding, the same study coordinator was designated throughout this study.

### 2.5. Statistical Analyses

Continuous variables are presented as mean ± SD and categorical variables as absolute number and percentage. Spearman's rank correlation test was performed to measure how closely the COPD assessed by CCQ score agreed with that by GOLD classification and that by the BODE index. Comparison of mean CCQ scores/CCQ domain scores in different GOLD stages and BODE index quartiles was performed by one-way ANOVA. A 2-sided value of *P* < 0.05 was considered statistically significant. Statistical analyses were performed using the SPSS software package, version 11.5 (SPSS Inc., Chicago, IL, USA).

## 3. Results

### 3.1. Characteristics of Study Subjects, Severity of COPD Measured with FEV1, FEV1/FVC, BODE Index Score, and CCQ Scores

A total of 89 clinically stable COPD patients were included for analyses (Table 1). The severity of COPD by GOLD classification, BODE index, and BODE quartiles of the included patients are detailed in Table 2. The CCQ total score, CCQ domain scores, and CCQ item scores are shown in Table 3.

### 3.2. Correlations of CCQ Scores and the Stratified COPD Severities by GOLD Criteria

**Figure 1:**
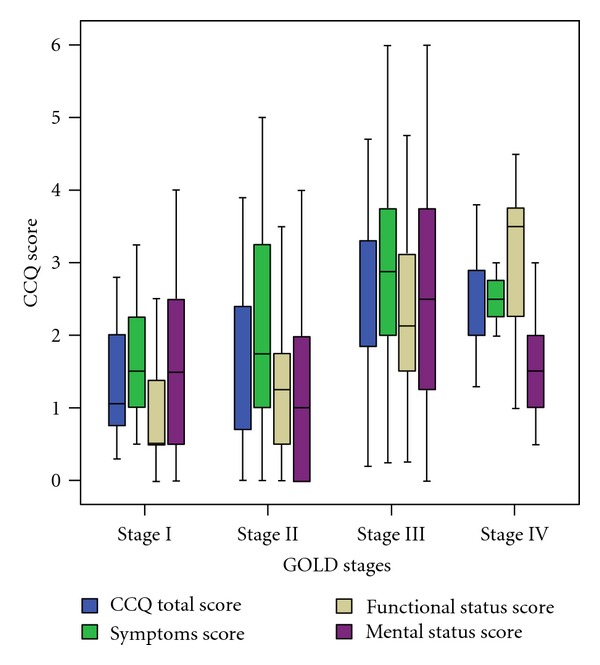
Box plot for CCQ scores by GOLD classification (FEV1 % predicted). Error bars show standard deviation and horizontal lines within boxes show the mean for CCQ scores.

Statistically significant correlation was found between the CCQ total scores/CCQ function scores and the stratified COPD severities (*P* < 0.001), between CCQ symptom score and the stratified COPD severities (*P* = 0.001), and between CCQ mental score and the stratified COPD severities (*P* = 0.03) (Figure 1). Mean CCQ total scores/mean CCQ domain scores corresponding to the stratified COPD severities (stages I–IV) by GOLD criteria were as follows. Mean CCQ total scores were 1.33 (stage I), 1.58 (stage II), 2.63 (stage III), and 2.54 (stage IV). Mean CCQ function scores were 0.9 (stage I), 1.26 (stage II), 2.38 (stage III), and 3.00 (stage IV). Mean CCQ symptom scores were 1.67 (stage I), 2.07 (stage II), 2.91 (stage III), and 2.64 (stage IV). Mean CCQ mental scores were 1.54 (stage I), 1.22 (stage II), 2.58 (stage III), and 1.44 (stage IV).

### 3.3. Correlation of CCQ Scores and COPD Severity by the BODE Index

**Figure 2:**
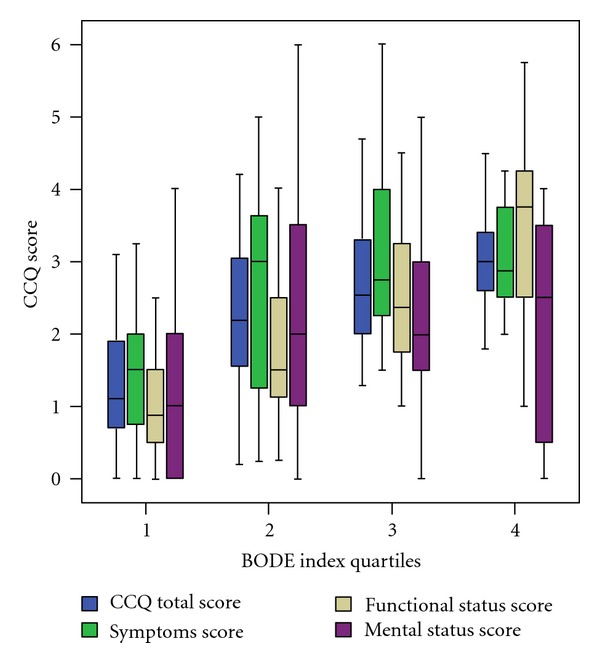
Box plot for CCQ scores by BODE quartiles. Error bars show standard deviation and horizontal lines within boxes show the mean for CCQ scores.

Statistically significant correlation was found between the CCQ total scores/CCQ function scores/CCQ symptom scores and the COPD severity by the BODE index (*P* < 0.001) and between CCQ mental scores and BODE quartiles (*P* = 0.001) (Figure 2). The mean CCQ total scores/CCQ domain scores corresponding to the BODE quartiles (quartiles I-IV) were as follows. Mean CCQ total scores were 1.33 (quartile I), 2.26 (quartile II), 2.78 (quartile III), and 3.1 (quartile IV). Mean CCQ function scores were 1.00 (quartile I), 1.81 (quartile II), 2.60 (quartile III),  and 3.60 (quartile IV). Mean CCQ symptom scores were 1.74 (quartile I), 2.62 (quartile II), 3.21 (quartile III), and 3.60 (quartile IV).

### 3.4. Correlation between CCQ Score and the BODE Index/GOLD Staging

The correlations of the total CCQ scores/CCQ domain scores with the BODE index and GOLD classification in assessment of severity of COPD and the Spearman correlation coefficients (*rS*) are detailed in Table 4. The CCQ functional scores correlated most with the BODE index (*rS* = 0.670) and GOLD classification (*rS* = 0.531), followed by the CCQ total scores. The CCQ total scores and each CCQ domain score were better correlated with the BODE index than with GOLD classification. 

## 4. Discussion

Our data demonstrated that CCQ scores had a good correlation with the BODE index and GOLD classification in assessing the severity of COPD. The robust correlation between CCQ scores and the BODE index, a documented practical instrument in predicting the severity and mortality in this patient population, suggests that it is reasonable to use CCQ as an alternative in the assessment of life quality of COPD patients.

When it comes to the correlation strength with BODE score, the CCQ domain scores/CCQ total score in decreasing order were CCQ functional score (*rS* = 0.670), CCQ total score (*rS* = 0.610), CCQ symptom score (*rS* = 0.482), and CCQ mental score (*rS* = 0.340). The CCQ functional domain aims at assessing limitations of activities because of the breathing problems, which includes four items: CCQ7 (strenuous physical activities), CCQ8 (moderate physical activities), CCQ9 (daily activities at home), and CCQ10 (social activities) (see the appendix for details). CCQ7-9 describes the extent of breathing problem during different degrees of activity, representing not only a patient's respiratory condition (FEV1), perception (MMRC), exercise capacity (6MWD). Of note, CCQ10 is a surrogate of complex parameters not only for physical activities, but also for socioeconomic activities; it is therefore not surprising that CCQ10 was correlated with MMRC, FEV1, and BMI.

The CCQ mental domain includes 2 items, namely, CCQ3 (concern about getting a cold or breathing getting worse?) and CCQ4 (depression because of breathing problems?), and, when comparing to other CCQ domains, CCQ mental domain was found to have weaker correlation with BODE index and GOLD classification. The CCQ mental score reflects psychological and subjective feelings of the patient, which are developed based on the personal experience, socioeconomic status, and attitude toward life. COPD is a systemic disease that is associated with systemic inflammation and skeletal muscle dysfunction [28–31]. These systemic effects lead to decline in health status, exercise de-conditioning, relative social isolation, and development of depressive mood, muscle wasting, and weight loss in the patients with COPD. The above-mentioned adverse factors form a vicious cycle in which depression is possibly further aggravated.

Because the complexity of CODP expresses itself in respiratory, perceptive, and systemic aspects [2], it is impossible to use the conventionally employed physiological variable FEV1 alone to depict each perspective and measure the severity of COPD. For example, FEV1 is weakly correlated with the degree of dyspnea [3], and the change in FEV1 fails to reflect the rate of decline in patients' health status [4]. The degrees of dyspnea [5] and health status scores [6] were reported to be more accurate than FEV1 in predicting the risk for death in patients with COPD. Therefore, new GOLD guidelines were announced in the annual meeting of Asia-Pacific Society of Respirology in November 2011 in Shanghai and suggested the assessment of the severity of COPD not only by FEV1 alone, but also by symptom scoring (e.g., MMRC dyspnea scale or CAT score) and the frequency of acute exacerbation of COPD. These conceptual changes in the GOLD guidelines underscore the important role of a good questionnaire in assessing the severity of COPD in clinical practice.

BODE score has a high value in evaluating COPD severity and predicting mortality of the affected patients [2]. However, calculation of BODE index requires collection of information regarding body weight as well as body length and data generated from spirometric and 6-minute walking tests. Spirometric and 6-minute walking tests are relatively time consuming, comparatively costly, and require the necessary equipments and involvement of qualified staff (e.g., chest physician and technician). As a result, the applicability of BODE index sometimes has been limited by the widespread resource-deficiency settings such as practitioner's clinics and facilities situated in remote rural areas. In the scenario that collection of data for calculation of BODE is possible, it is inconvenient for the patient in question because it is not until the following visit will he/she know the results of BODE assessment. In contrast, assessment of clinical severity of COPD with CCQ needs neither data generated from time-consuming process nor labor-intensive tests. CCQ is easy to complete by the patients themselves at their outpatient visits. Furthermore, the convenience of applicability of serial CCQ in follow-up evaluation makes detection of changes in severity of COPD more rapidly and effectively, and thus necessary intervention may start in a more timely fashion. However, this does not imply that CCQ can substitute BODE.

Some limitations in our study should be addressed. First, this study is only a cross-section study; longitudinal follow-up studies on outcomes concerning the frequencies of subsequent hospitalization and COPD acute exacerbation and mortality are needed to better measure the correlation between CCQ and the BDE index. Second, the numbers of patients included in this study were small and the patients are of the same race; COPD patients of different races may have different outcomes. Third, our data might be biased by the single medical-center-based patients rather than population-based patients included.

In conclusion, CCQ scores have a good correlation with BODE index and GOLD staging and CCQ scores correlate better with BODE index than GOLD staging in our cross-sectional study. However, whether CCQ score correlates with BODE index in a longitudinal study or nonhospital-based population or not needs further investigations.

## Figures and Tables

**Table 1:** Characteristics of 89 included men with clinically stable chronic obstructive pulmonary disease.

Characteristic	Mean ± SD
Age (yr)	70.6 ± 10.1
Male (%)	80.1 ± 0.2
Smoking history (pack-yr)	51.8 ± 28.2
FVC* (liter)	70.9 ± 18.9
FEV1/FVC^†^	55.4 ± 11.9
FEV1 (% of predicted value)	54.2 ± 21.7
MMRC dyspnea scale^‡^	1.9 ± 1.4
Distance walked in 6 min (m)	398.4 ± 115.2
Body mass index^§^	23.4 ± 3.5

*FVC denotes forced vital capacity, and ^†^FEV1 forced expiratory volume in one second. ^‡^Scores on the modified Medical Research Council (MMRC) dyspnea scale may range from 0 to 4, with a score of 4 indicating that the patient is too breathless to leave the house or becomes breathless when dressing or undressing. ^§^The body mass index is the weight in kilograms divided by the square of the height in meter.

**Table 2:** GOLD stages of chronic obstructive pulmonary disease (COPD), BODE index score, and BODE quartiles in the 89 COPD patients.

Characteristic	Number of patients (%)
COPD GOLD stage	
Stage I (FEV1* > 80% of predicted value)	12 (13.5)
Stage II (FEV1, 50–80% of predicted value)	36 (40.4)
Stage III (FEV1, 30–50% of predicted value)	32 (36)
Stage IV (FEV1 ≤ 30% of predicted value)	9 (10.1)

BODE index^†^	
0	15 (16.9)
1	16 (18.0)
2	11 (12.4)
3	10 (11.2)
4	9 (10.1)
5	12 (13.5)
6	6 (6.7)
7	6 (6.7)
8	2 (2.2)
9	2 (2.2)
10	0 (0)

BODE quartile^‡^	
1	42 (47.2)
2	19 (21.3)
3	18 (20.2)
4	10 (11.2)

*FEV1 denotes forced expiratory volume in one second. ^†^See text for details of BODE; the higher the BODE index, the greater the risk of death. ^‡^Quartile 1 referred to BODE score of 0 to 2, quartile 2 to 3 to 4, quartile 3 to 5 to 6, and quartile 4 to 7 to 10.

**Table 3:** Scores of Clinical COPD Questionnaire score (CCQ) and those of its components in 89 patients with clinically stable chronic obstructive pulmonary disease.

Variable	Score (mean ± SD)
CCQ item*	
CCQ1	1.16 ± 1.36
CCQ2	3.30 ± 2.03
CCQ3	1.93 ± 1.70
CCQ4	1.62 ± 1.50
CCQ5	2.58 ± 1.81
CCQ6	2.46 ± 1.68
CCQ7	2.89 ± 1.58
CCQ8	2.43 ± 1.63
CCQ9	1.26 ± 1.51
CCQ10	0.58 ± 1.05

CCQ domain	
Symptoms	2.38 ± 1.37
Functional status	1.79 ± 1.31
Mental status	1.78 ± 1.49
CCQ total score	2.02 ± 1.16

*An individual CCQ item refers to one individual question in CCQ (see appendix for details), and these items were allocated in one of the three CCQ domains as follows: symptoms (items 1, 2, 5, and 6), functional state (items 7, 8, 9, and 10), and mental state (items 3 and 4).

**Table 4:** Spearman correlation between CCQ scores and the BODE index/GOLD classification in assessment of severity of COPD.

CCQ	BODE index		GOLD classification	
	Correlation coefficient (*rS*)	*P*	Correlation coefficient (*rS*)	*P*
CCQ symptom scores	0.482	<0.001	0.346	0.001
CCQ functional scores	0.670	<0.001	0.531	<0.001
CCQ mental scores	0.340	0.001	0.236	0.026
CCQ total scores	0.610	<0.001	0.462	<0.001

## Appendix

The Clinical COPD Questionnaire (CCQ) [18] contains 10 items (CCQn, where n is the numeric order ranging from 1–10). Patients were instructed to recall their experiences during the p previous week (week-version CCQ) regarding on average, how often did they feel short of breath at rest? (CCQ1), short of breath doing physical activities? (CCQ2), concerned about getting a cold or breathing problems? (CCQ3), and depressed (down) because of breathing problems? (CCQ4); in general, how much of the time did they cough? (CCQ5), and did they produce phlegm? (CCQ6); on average, how limited were they in the following activities because of their breathing problems: strenuous physical activities (CCQ7), moderate physical activities (CCQ8), daily activities at home (CCQ9), and social activities (CCQ10). Each patient responded to each of CCQn with a 7-point scale ranging from 0 (indicating asymptomatic/no limitation) to 6 (indicating extremely symptomatic/totally limited). Calculation of scores were as follows: CCQ total score = (CCQ1 + 2 + 3 + 4 + 5 + 6 + 7 + 8 + 9 + 10)/10; CCQ symptom score = (CCQ1 + 2 + 5 + 6)/4; CCQ function score = (CCQ7 + 8 + 9 + 10)/4; CCQ mental state score = (CCQ3 + 4)/2.

## Abbreviations

FEV1: Forced expiratory volume in the first second
CCQ: The Clinical COPD Questionnaire
COPD: Chronic obstructive pulmonary disease
BODE: Body mass index (B), the degree of airflow obstruction (O), functional dyspnea (D), and exercise capacity (E)
MMRC: The modified Medical Research Council
6MWD: The distances of the six-minute-walk test.
